# A surprising twist

**DOI:** 10.7554/eLife.02715

**Published:** 2014-04-08

**Authors:** Nikta Fakhri, Christoph F Schmidt

**Affiliations:** 1 **Nikta Fakhri** is in the Faculty for Physics–Third Institute of Physics–Biophysics, Georg August University, Göttingen, Germany; 2 **Christoph F Schmidt** is in the Faculty for Physics–Third Institute of Physics–Biophysics, Georg August University, Göttingen, Germanychristoph.schmidt@phys.uni-goettingen.de

**Keywords:** mitosis, kinesin-5, microtubule, motor proteins, coiled-coil, x-ray structure, *D. melanogaster*

## Abstract

X-ray crystallography has revealed an unusual structural element in kinesin-5 motor proteins.

**Related research article** Scholey JE, Nithianantham S, Scholey JM, Al-Bassam J. 2014. Structural basis for the assembly of the mitotic motor kinesin-5 into bipolar tetramers. *eLife* **3**:e02217. doi: 10.7554/eLife.02217

**Image** Kinesin-5 motor proteins move antiparallel microtubules

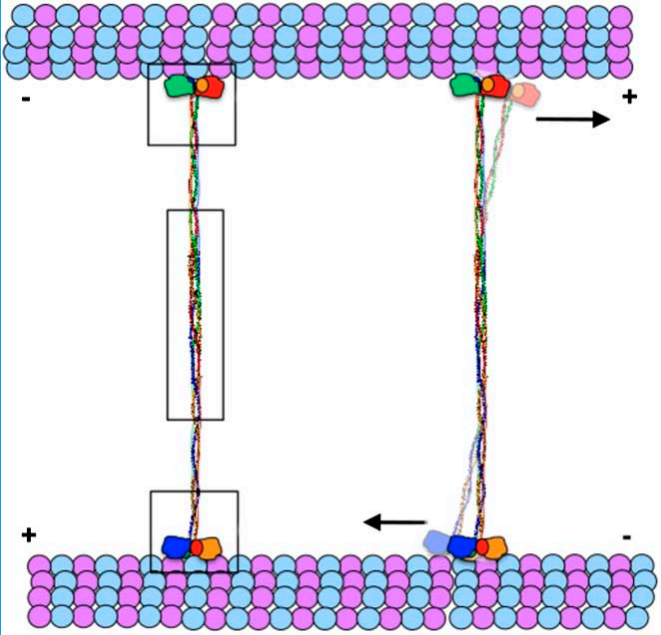

Cells contain tiny biological machines called motor proteins that move cargoes along tracks called microtubules, and in some cases also move the microtubules themselves. This is particularly crucial during cell division, where the microtubules build the cellular machine, called the mitotic spindle, that ensures that each daughter cell receives the correct number of chromosomes (Sawin et al., 1992).

A family of motor proteins called kinesin-5 is responsible for the movement of the microtubules. Kinesin-5 proteins are shaped like a dumbell in which two pairs of ‘heads’ are attached to a central stalk: a closer look reveals that the functional motor is actually a tetramer made up of four identical subunits (Figure 1). Each pair of heads is able to move along a microtubule in a defined direction, and by working together they can slide two microtubules apart if the microtubules are antiparallel (Gheber et al., 1999; Kapitein et al., 2005).

**Figure 1.**
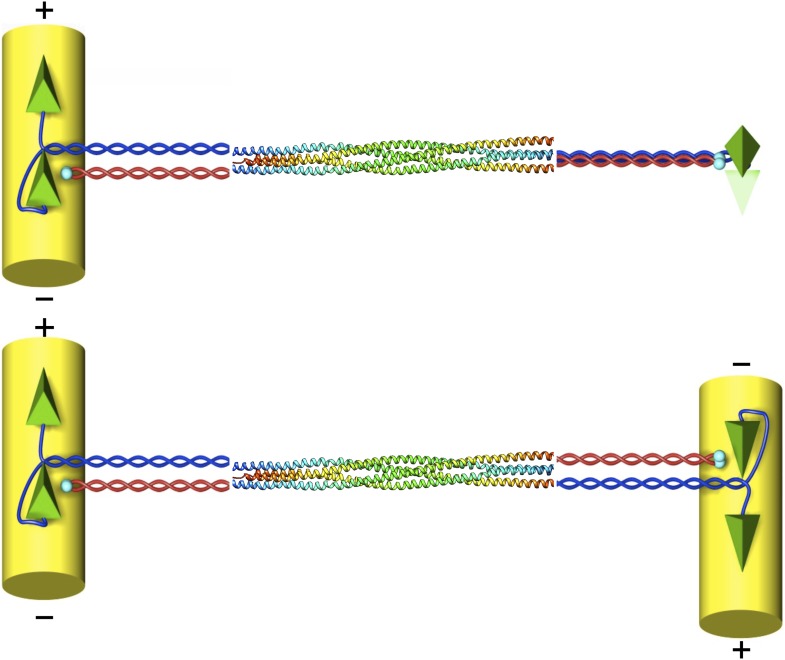
The structure of the kinesin-5 motor protein. Kinesin-5 is a tetramer that contains four α-helices (blue and red) that end at ‘heads’ (green pyramids) or ‘tails’ (cyan spheres). Pairs of heads move along microtubules (yellow cylinders) towards the plus end. The α-helices intertwine in a parallel coiled coil structure, and they swap partners in the BASS domain, which plays a key role in making the motor protein work. Scholey et al. have determined the structure of the BASS domain (shown here by the multicoloured helices, which are taken from Figure 2 of Scholey et al., 2014). When kinesin-5 is attached to only one microtubule (top), the structure of the BASS domain discovered by Scholey et al. predicts that the two ends of the tetramer are rotated by 90°. When kinesin-5 motors slide antiparallel microtubules apart (bottom), the motor domains must also be antiparallel to each other. The resulting torque in the attached tetramer might help to flag the mutual binding state and turn the motor on. The twist that would have to occur in the tetramer in this situation is not shown as it is not known where it would be localised.

Although the atomic structure of the head of kinesin-5 has been worked out (Turner et al., 2001), the structure of the stalk has not. This is primarily because it turned out to be difficult to crystallize and, possibly, because researchers expected it to be a rather boring α-helical coiled coil. However, the many roles that the stalk performs require a more complex structure. The stalk needs to transmit tensile force between the two pairs of heads when they move apart on their respective microtubule tracks. It also needs to provide torsional rigidity to orient the heads properly (van den Wildenberg et al., 2008). Last, but not least, it needs to be able to convey mechanical signals between the two heads. Now, in *eLife*, Jawdat Al-Bassam of the University of California, Davis and co-workers—Jessica Scholey and Stanley Nithianantham, as joint first authors, and Jon Scholey—report that the central part of the stalk of a kinesin-5 motor contains an unexpected and intriguing structural element called a bipolar assembly (BASS) domain (Scholey et al., 2014).

Last year a collaboration led by Jon Scholey identified the limits of the BASS domain and found that it is necessary to assemble the antiparallel tetramers (Acar et al., 2013). Now Al-Bassam and co-workers have succeeded in crystallizing this region taken from a kinesin-5 found in *Drosophila*, and have analysed its atomic structure using X-ray crystallography.

What was highly surprising about the structure they found was that in the BASS domain, the two parallel coiled coils extending from each pair of motor heads change into two antiparallel coiled coils. The α-helices of the motors switch partners, jumping from a parallel partner coil to an antiparallel partner coil at precisely defined locations to again form a tetramer. The two opposing coils in a pair, and the two pairs themselves, interact by a precisely engineered pattern of seven alternating hydrophobic and ionic patches over a record length of 26 nm: this is the longest helical minifilament structure determined to date. Mutational analysis shows that the precise arrangement of these patches is necessary to form the tetramer.

This structure provides strong mechanical coupling between the two heads, and a strong resistance to tensile forces. Tensile forces are, by the way, the typical load that the motors experience, whether during the process of pushing microtubules apart against an external load, or resisting an external force pulling the microtubules apart. Compressional forces immediately lead to the stalks buckling.

Another rather intriguing structural feature of the BASS domain is that the two dimers in the stalk emerge with a 90° relative twist after swapping partners and then rejoining their original partner again. This implies that the heads would also be twisted by 90° with respect to each other when viewed along the long axis of the tetramer (Figure 1). Scholey et al. speculate that this particular geometry, taken together with the expected high torsional stiffness of the whole structure, might explain why kinesin-5 prefers to bundle and move antiparallel microtubules.

Like the whole process of cell division, the activity of kinesin-5 motors must be tightly regulated as the cell divides to make sure that nothing goes wrong. Some regulatory mechanisms have been observed. It was found, for example, that certain kinesin-5 motors only become active and move when both ends are connected to a microtubule, and only when those two microtubules are antiparallel (Kapitein et al., 2008). Even more intriguingly, a kinesin-5 from budding yeast switches its direction of motion when reaching an antiparallel overlap zone (Gerson-Gurwitz et al., 2011; Roostalu et al., 2011). It is therefore tempting to speculate further on the ways that this motor protein works.

Once the heads of kinesin-5 are moving along antiparallel microtubules, they will have to be oriented antiparallel to each other (Figure 1). This should cause torsional stress and strain that might serve as a mechanism to turn on the motors or, in the case of the yeast, to change direction. Since torsional stress can be transmitted even along a rod that is flexible to bending, as seen in the cable of a dentist’s drill, such a mechanism could explain how signalling occurs over the extraordinarily long distances seen in the kinesin-5 motors. If this model is correct, it leads to an interesting prediction: when two microtubules are connected by a single kinesin-5 motor they should spontaneously orient at 90°. If an external force is then used to change this angle to 180°, the motor should turn on. This prediction could be tested in experiments that use optical tweezers to apply the external force.

The crossing over of the coiled-coils in the BASS domain also suggests a kinetic mechanism for the formation of the tetramer. If the antiparallel coupling of two motor monomers is rapid and strong, and is then followed by two of those dimers joining into a tetramer, it would explain why the formation of tetramers is so efficient. It would also explain why a large sub-population of parallel dimers is not also created. Such dynamic consequences of the newly discovered surprising structure of the motor stalks will have to be explored in future experiments.
